# Soot as a Deodorizer of Privies

**Published:** 1853-11

**Authors:** Robert Elliot

**Affiliations:** Carlisle


From the London Lancet.

*Soot as a Deodorizer of Privies.*

We in Carlisle (on the Local Board of Health) have derived great help, at little cost, in the removal of manure, otherwise so perilous, by the immediately subsequent use of a few shovelfuls of soot. The suggestion was made to us by the Rev. Mr. Dew, of St. Cuthbert's Church, and serves to show what important aid may just now be rendered by the co-operation of the clergy and other intelligent men. Soot is, generally, most easily had where quicklime is scarce, and *vice-versa.* In Carlisle, British cholera prevails abundantly and obstinately; but with the exception of one case that ended fatally, and, on the evidence of two intelligent and experienced medical men, exhibited unequivocally the symptoms of Asiatic cholera, we have had no clearly marked cholera here. I know of no other cases of death from this or similar diseases at this juncture, except the single one mentioned, and this was a fortnight ago. Carlisle is abundantly supplied with pure river water (from far above the city), and our common lodging-houses have long been most carefully regulated. Our operative and poorer classes, too, have been long, and are undergoing a process of spreading out, or popular evolution, which, by giving to each family their own door and more elbow-room, is clearly productive of immense good. Between our ancient city and the equally old town of Newcastle-on-Tyne there is daily railway communication, by five trains each way. Cholera rages but sixty miles off, in Newcastle, more or less since the 31st ult.; it has latterly been slaying its five or six scores daily, yet has not in three weeks got possession of Carlisle, though cheap trips, with goodly numbers of passengers, pass both east and west. Let the advocates of contagion or infection (the idea of which is too paralyzing to the non-medical community—too natural to the timid and uninformed, to need encouragement)—let them examine these facts, and do the fullest justice to them, since an opposite course is so palpably unfair, unmanly, and impolitic. An old Indian army surgeon and his lady, have just told me that the natives have no such ideas.

ROBERT ELLIOT, M.D.

Carlisle, Sept., 1853.

